# Supplementary material for: Laser Ablated Periodic Nanostructures on Titanium and Steel Implants Influence Adhesion and Osteogenic Differentiation of Mesenchymal Stem Cells
Source: Materials (Basel). 2020 Aug 10;13(16):3526. doi: 10.3390/ma13163526 (PMC7475978; doi:10.3390/ma13163526)
Supplement: Supplementary file 1 [file materials-13-03526-s001.pdf]

materials

Supplementary Materials

Laser Ablated Periodic Nanostructures on Titanium and Steel Implants Influence Adhesion and Osteogenic Differentiation of Mesenchymal Stem Cells

Kai Oliver Böker [1,*], Frederick Kleinwort [2], Jan-Hendrick Klein-Wiele [2], Peter Simon [2], Katharina Jäckle [1], Shahed Taheri [1], Wolfgang Lehmann [1] and Arndt F. Schilling [1]

[1] Department for Trauma Surgery, Orthopaedics and Plastic Surgery, University Medical Center Goettingen, Robert Koch Straße 40, 37075 Göttingen, Germany; katharina.jaeckle@med.uni-goettingen.de (K.J.); shahed.taheri@med.uni-goettingen.de (S.T.); Wolfgang.Lehmann@med.uni-goettingen.de (W.L.); arndt.schilling@med.uni-goettingen.de (A.F.S.)

[2] Laser-Laboratorium Göttingen e.V. (LLG), Hans-Adolf-Krebs-Weg 1, 37077 Göttingen, Germany; frederick.kleinwort@llg-ev.de (F.K.); jhkw@llg-ev.de (J.-H.K.-W.); peter.simon@llg-ev.de (P.S.)

* Correspondence: kai.boeker@med.uni-goettingen.de; Tel.: +49-(0)-551-39-22613

Table S1: Linear structure depths D1-D5 on steel plates measured by AFM.

Structure	1-1	1-2	1-3	1-4	1-5
**Steel**	200 nm	360 nm	550 nm	710 nm	880 nm
**Titanium**	200 nm	320 nm	420 nm	530 nm	700 nm

Table S2: *Osteocalcin* statistical test.

Tukey's Multiple Comparison Test	Mean Diff.	q	Significant? $p < 0.05$?	Summary
Control plastic vs. Diff plastic	−1.624	7392	Yes	***
Control steel vs. Diff steel	−1.587	7574	Yes	***
Control steel vs. Diff steel 1-5	−0.2221	1011	No	ns
Control steel vs. Diff steel 2-5	−0.3903	1863	No	ns
Control steel vs. Diff steel 5-5	−0.5811	2734	No	ns
Control titanium vs. Diff titanium	−1.331	6056	Yes	**
Control titanium vs. Diff titanium 1-5	−0.07189	0.3382	No	ns
Control titanium vs. Diff titanium 2-5	−0.2168	0.9867	No	ns
Control titanium vs. Diff titanium 5-5	−0.3832	1803	No	ns

Table S3: *Collagen 1* statistical test.

Tukey's Multiple Comparison Test	Mean Diff.	q	Significant? $p < 0.05$?	Summary
Control plastic vs. Diff plastic	−0.6554	5.485	Yes	**
Control steel vs. Diff steel	−0.7635	6.702	Yes	***
Diff steel vs. Diff steel 1-5	0.5378	4.502	No	ns
Diff steel vs. Diff steel 2-5	0.6118	5.371	Yes	*
Diff steel vs. Diff steel 5-5	0.7835	6.779	Yes	***
Control titanium vs. Diff titanium	−0.4104	3.602	No	ns
Diff titanium vs. Diff titanium 1-5	0.4562	3.947	No	ns
Diff titanium vs. Diff titanium 2-5	0.3187	2.757	No	ns
Diff titanium vs. Diff titanium 5-5	0.3418	2.957	No	ns

Table S4: *RUNX2* statistical test.

Tukey's Multiple Comparison Test	Mean Diff.	q	Significant? $p < 0.05$?	Summary
Control plastic vs. Diff plastic	−1.583	6.298	Yes	**
Control steel vs. Diff steel	−2.000	8.203	Yes	***
Diff steel vs. Diff steel 1-5	1.703	6.658	Yes	***
Diff steel vs. Diff steel 2-5	1.708	7.005	Yes	***
Diff steel vs. Diff steel 5-5	1.711	6.915	Yes	***
Control titanium vs. Diff titanium	−1.530	6.276	Yes	**
Diff titanium vs. Diff titanium 1-5	1.239	5.010	Yes	*
Diff titanium vs. Diff titanium 2-5	0.5775	2.258	No	ns
Diff titanium vs. Diff titanium 5-5	1.142	4.618	No	ns

Table S5: *Aggrecan* statistical test.

Tukey's Multiple Comparison Test	Mean Diff.	q	Significant? $p < 0.05$?	Summary
Control plastic vs. Diff plastic	0.6275	6.947	Yes	***
Control steel vs. Diff steel	0.8696	1.010	Yes	***
Diff steel vs. Diff steel 1-5	0.06209	0.6874	No	ns
Diff steel vs. Diff steel 2-5	0.09927	1.153	No	ns
Diff steel vs. Diff steel 5-5	0.01721	0.1969	No	ns
Control titanium vs. Diff titanium	1.178	1.367	Yes	***
Diff titanium vs. Diff titanium 1-5	−0.1279	1.464	No	ns
Diff titanium vs. Diff titanium 2-5	−0.1929	2.135	No	ns
Diff titanium vs. Diff titanium 5-5	0.05360	0.6224	No	ns

Table S6: *Collagen 2* statistical test.

Tukey's Multiple Comparison Test	Mean Diff.	q	Significant? $p < 0.05$?	Summary
Control plastic vs. Diff plastic	0.4287	4.406	No	ns
Control steel vs. Diff steel	0.2925	3.153	No	ns
Diff steel vs. Diff steel 1-5	0.1781	1.831	No	ns
Diff steel vs. Diff steel 2-5	0.1885	2.032	No	ns
Diff steel vs. Diff steel 5-5	0.08937	0.9494	No	ns
Control titanium vs. Diff titanium	0.2760	2.836	No	ns
Diff titanium vs. Diff titanium 1-5	−0.3775	4.010	No	ns
Diff titanium vs. Diff titanium 2-5	−0.5897	6.060	No	ns
Diff titanium vs. Diff titanium 5-5	−0.09301	0.9880	No	ns

Table S7: *Sox9* statistical test.

Tukey's Multiple Comparison Test	Mean Diff.	q	Significant? $p < 0.05$?	Summary
Control plastic vs. Diff plastic	−0.3593	5.499	Yes	**
Control plastic vs. Control steel	−0.07352	1.125	No	ns
Control steel vs. Diff steel	−0.1894	2.897	No	ns
Diff steel vs. Diff steel 1-5	0.03785	0.552	No	ns
Diff steel vs. Diff steel 2-5	0.1487	2.276	No	ns
Diff steel vs. Diff steel 5-5	0.03908	0.589	No	ns
Control titanium vs. Diff titanium	−0.1630	2.494	No	ns

Diff titanium vs. Diff titanium 1-5	0.1778	2.681	No	ns
Diff titanium vs. Diff titanium 2-5	−0.07077	1.013	No	ns
Diff titanium vs. Diff titanium 5-5	−0.1728	2.606	No	ns

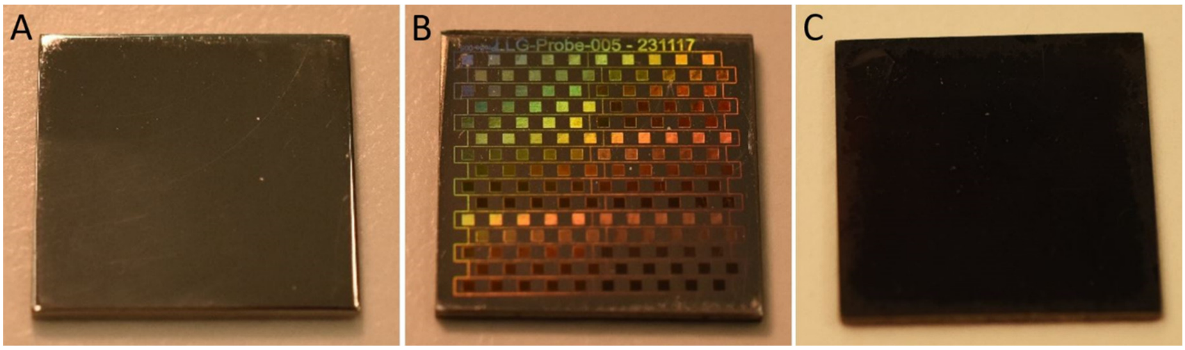

Figure S1: Representation of non-treated plates (**A**), laser-structured plates with 30 different structure types and depths (**B**), and fully structured plates (**C**).

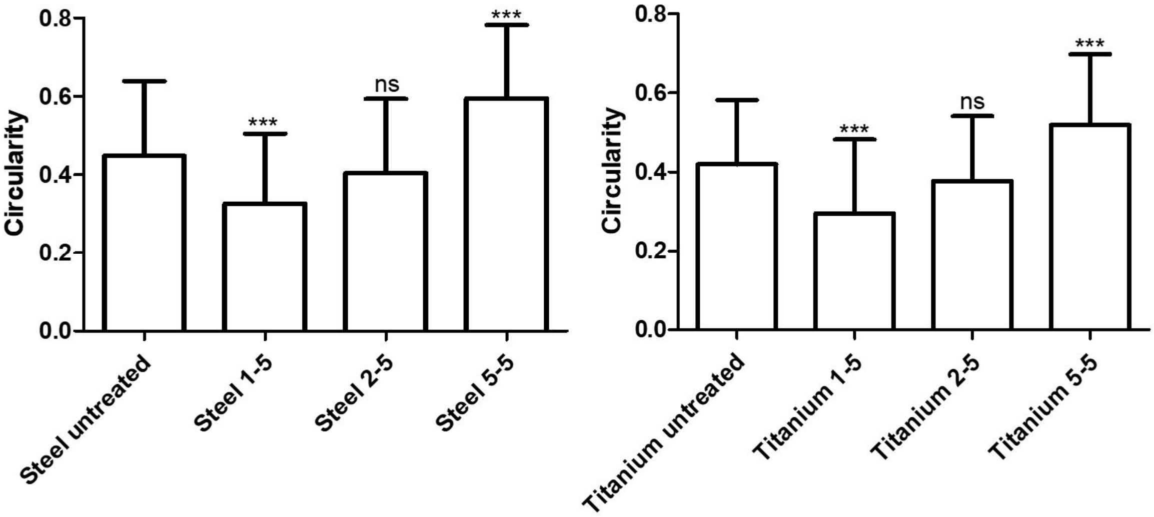

Figure S2: Circularity of SCP1 cells on different surfaces. Structure 1-5 decreased the circularity of cells on titanium and steel plates compared to non-structured control surfaces while structure 5-5 increased circularity. A circularity of 1.0 indicates a perfect circle, while a circularity closer to 0.0 indicates an increasingly elongated polygon.

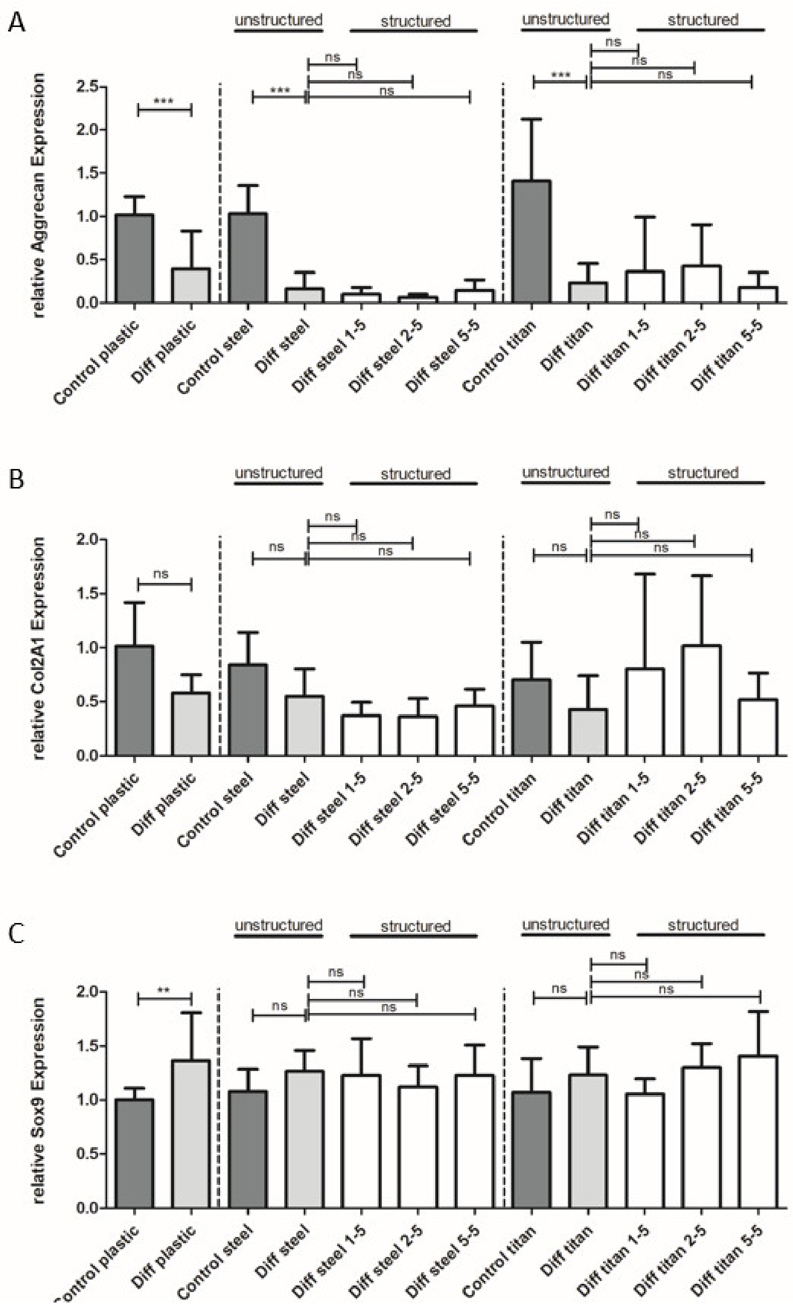

Figure S3: The influence of plate structure on chondrogenic genes after osteogenic differentiation. Gene expression analysis of chondrogenic genes was performed after 4 weeks of differentiation of human mesenchymal stem cells. *Aggrecan* expression (**A**) was reduced upon osteogenic differentiation on polystyrene, steel, and titanium plates, while plate structuring showed no difference on *Aggrecan* expression. *Collagen 2* expression (**B**) was not affected upon osteogenic differentiation, neither by the surface type (polystyrene, steel, titanium), nor through structuring. *Sox9* expression (C) was increased by osteogenic differentiation on polystyrene surfaces, while no change was observed on steel or titanium plates. ** ($p < 0.005$), *** ($p < 0.0005$), ns (non-significant).
